# Correlation between neuropsychiatric symptoms and caregiver burden in a population-based sample from São Paulo, Brazil: a preliminary report

**DOI:** 10.1590/S1980-57642013DN70300005

**Published:** 2013

**Authors:** Jefferson Cunha Folquitto, Rita de Cássia Gomes Marques, Mariana Franciosi Tatsch, Cássio Machado de Campos Bottino

**Affiliations:** 1 Old Age Research Group, Institute of Psychiatry, University of São Paulo, São Paulo, Brazil.

**Keywords:** Alzheimer's disease, cognitive impairment no dementia, neuropsychiatric symptoms, caregiver burden

## Abstract

Neuropsychiatric symptoms and caregiver burden are highly prevalent in older adults with Alzheimer's disease (AD).OBJECTIVE: To evaluate the correlation between neuropsychiatric symptoms and caregiver burden in a community-based sample from São Paulo, Brazil.

**METHODS:**

A total of 1,563 randomly-selected subjects were assessed by the Mini-Mental State Examination, Fuld Object Memory Evaluation, Informant Questionnaire on Cognitive Decline in the Elderly and Bayer – Activities of Daily Living Scale. Subjects considered screen-positives were submitted to a dementia workup and diagnosis was determined according to ICD-10 criteria. The neuropsychiatric Inventory was applied to caregivers to evaluate neuropsychiatric symptoms and the Zarit Burden Interview was also applied to assess caregivers' burden.

**RESULTS:**

Sixty-one AD patients, 25 Cognitively Impaired Non Demented (CIND) and 79 healthy elderly subjects were evaluated. Zarit mean scores for controls, CIND and AD were 2.32, 3.92 and 20.11, respectively. There was strong positive correlation between total NPI and Zarit scores.

**CONCLUSION:**

In conclusion, neuropsychiatric symptoms showed a significant association with higher rates of caregiver stress.

## Abstract (pt)

Sintomas neuropsiquiátricos e sobrecarga do cuidador apresentam alta prevalência em idosos com doença de Alzheimer (DA).

**OBJETIVO:**

Avaliar a correlação entre sintomas neuropsiquiátricos e sobrecarga do cuidador em uma amostra comunitária de São Paulo, Brasil.

**MÉTODOS:**

Um total de 1.563 indivíduos selecionados aleatóriamente foram avaliados através do Mini-Exame do Estado Mental, *"Fuld Object Memory Evaluation", "Informant Questionnaire on Cognitive Decline in the Elderly" *e da Escala Bayer – Atividades de Vida Diária. Indivíduos considerados suspeitos foram submetidos a uma investigação para demência, sendo o diagnóstico feito com os critérios da CID-10. Inventário Neuropsiquiátrico foi aplicado nos cuidadores para avaliar sintomas neuropsiquiátricos e a Escala Zarit de Sobrecarga no Cuidador para avaliar sobrecarga nos cuidadores.

**RESULTADOS:**

Sessenta e um pacientes com DA, 25 sujeitos com Comprometimento Cognitivo não Demência (CIND) e 79 idosos saudáveis foram avaliados. A média dos escores da Zarit para controles, CIND e DA foi, respectivamente, 2,32, 3,92 e 20,11 pontos. Houve correlação positiva boa entre os escores da NPI e da Zarit.

**CONCLUSÃO:**

Sintomas neuropsiquiátricos mostraram uma associação significativa com escores mais elevados de estresse do cuidador.

## INTRODUCTION

Neuropsychiatric symptoms are highly prevalent, affecting 10% to 73% of dementia patients,^1^ with this variation being due to differences in assessment methods and in population samples studied.^2^ The neuropsychiatric symptoms include delusions, hallucinations, agitation (physical and verbal), depression, anxiety, euphoria/elation, apathy, disinhibition, irritability, aberrant motor behavior, night-time disturbances and eating changes.^2^ In Brazil, Tatsch et al.^2^ found a prevalence of neuropsychiatric symptoms in AD and CIND (cognitive impairment no dementia) of 78.3% and 36%, respectively. The most frequent symptoms in AD were apathy (53.3%), depression (38.3%), sleep disturbances (38.3%) and anxiety (25%), whereas in CIND these were anxiety (24%), night-time disturbances (24%) and depression (16%). In a comprehensive literature review, apathy and depression were considered the most prevalent neuropsychiatric symptoms in subjects with AD and CIND.^3^

Neuropsychiatric symptoms in patients with dementia are associated with worse prognoses, higher health care costs, greater impairment in daily functioning and quality of life, faster cognitive decline, earlier institutionalization, higher mortality, and increased caregiver burden.^2-4^ These serious consequences call for the development of new strategies for the prevention, early recognition and intervention to deal with neuropsychiatric symptoms in dementia.^3^

A caregiver can be defined as a person who helps with the basic and daily instrumental activities of daily living of a patient for most of the time, without receiving payment for this activity.^5^ Caregiver burden is defined as the sum of physical, psychological, social and financial problems which arise among members of the family or people who assist the diseased elderly.^6^

The presence of neuropsychiatric symptoms in patients with dementia and subjects with mild cognitive impairment is associated with greater caregiver burden.^7^ The symptoms that more frequently cause caregiver burden are aggression and delusions.^8^

The aim of the present study was to evaluate the influence of neuropsychiatric symptoms on caregiver burden in a community-based sample of elderly subjects with Alzheimer's disease or cognitive impairment no dementia (CIND).

## METHODS

The present study evaluated subjects aged 60 years or over from an epidemiological survey conducted in São Paulo, Brazil. In the first phase, a total of 1,563 elderly, drawn from three districts of the urban area of São Paulo representing high, medium and low socioeconomic classes, was evaluated. The following screening algorithm was applied to identify subjects suspected of being demented: Mini-Mental State Examination (MMSE)^9^ scores for illiterate <20; 1-4 years of schooling <25. 5-8 years of schooling <27; and ≥9 years of schooling <28; *or* Fuld Object Memory Evaluation score <35^10^ *and* Informant Questionnaire on Cognitive Decline in the Elderly^11^ score >3.40 *or* Bayer – Activities of Daily Living^12^ score >3.19. This screening method was previously tested in an outpatient sample of 93 elderly outpatients (34 AD and 59 controls), showing a sensitivity and specificity of 100%.^13^

In the second phase, screen positives were submitted to a work-up for dementia, entailing physical and neurological examination, Cranial Computed Tomography or Brain Magnetic Resonance Imaging, application of the Cognitive Section (CAMCOG) of the Cambridge Examination for mental Disorders (CAMDEX),^14,15^ Clinical Dementia Rating Scale (CDR),^16^ Neuropsychiatric Inventory (NPI)^17^ and Zarit Caregiver Burden Interview (which includes health of caregivers, psychological wellbeing, finances, social life and relationship between caregivers and patients. NPI and Zarit were applied using their standardized Brazilian versions.^18,19^

Diagnoses of Alzheimer's Disease (AD) and Cognitive Impairment No Dementia (CIND) were determined based on criteria of the DSM-IV^20^ and Ebly et al.,^21^ respectively.

This study included subjects with a diagnosis of dementia at the end of the second phase, and 79 elderly considered screen-negative in the first phase (randomized by SPSS 16.0 for Windows).

The study was approved by the Ethics Research Committee from the Clinicas Hospital of the Medical School of the University of São Paulo and all subjects evaluated gave consent to participate in the study.

Statistical analyses were performed using SPSS version 16.0 for Windows. For continuous variables, the Kolmogorov-Smirnov test was applied to test for normality. The Chi-square test was used to compare sex and the Kruskal-Wallis test to compare age and education. The Mann-Whitney test was applied to analyze the groups using two-by-two comparisons. The Spearman rank correlation coefficient was used to evaluate the correlation between NPI and Zarit.

## RESULTS

A total of one hundred and sixty-five caregivers were evaluated: 61 caregivers of patients with CIND, and 79 caregivers of normal elderly.

Table 1 shows the demographic characteristics of subjects and comparisons between the three groups.

**Table 1:** Demographic characteristics of sample.

Groups		Control	CIND	AD	Statistical analysis
Age	Mean	72.41	71.84	80.07^#,+^	*χ^2^=27.344
	(SD)	(8.16)	(9.25)	(8.84)	p<0.001
Education	Mean	643	3.71**	3.36^#^	*χ^2^=22.583
	(SD)	(5.01)	(4.74)	(4.49)	p<0.001
Sex	Male	24	9	14	χ^2^=1.757
	(%)	(30.45%)	(36.0%)	(23.0%)	p>0.05

SD: Standard Deviation;

* Kruskal-Wallis test among three groups;

** Mann-Whitney – controls versus CIND: U=522.500, p: 0.002;

# Mann-Whitney – controls versus AD: age (U=1254,000; p<0,001) and education (U=1140.500; p<0.001);

+ Mann-Whitney – CIND versus AD: U=375.500, p<0.001

Table 2 gives the number of subjects who had at least one symptom in each subsection and the mean Zarit scores for each NPI domain.

**Table 2:** Zarit scores according to each NPI domain.

Domains	Number of Subjects(Score: Item ≥ 1)	ZaritMean (SD)
Delusions	7	39.86 (25.73)
Hallucinations	5	34.00 (18.00)
Agitation	17	27.24 (25.06)
Depression	32	22.13 (19.06)
Anxiety	26	17.62 (17.45)
Euphoria	3	41.33 (26.63)
Apathy	35	20.71 (16.19)
Disinhibition	10	37.30 (24.66)
Irritability	18	25.22 (22.53)
Aberrant motor behavior	6	38.33 (21.23)
Night-time disturbance	32	21.91 (21.12)
Eating changes	15	24.33 (20.81)

Comparing the subjects by diagnostic group, there was a statistically significant difference, as evaluated using the Kruskal-Wallis test, for both total NPI score (Χ^2^=65.848; p<0.001) and Zarit score (Χ^2^=79.266; p<0.001), which were higher in the AD group. Mean scores on the NPI and Zarit by diagnostic group are depicted in Table 3.

**Table 3:** NPI and Zarit scores by diagnostic group.

	Controls Mean (SD)	CIND Mean (SD)	AD Mean (SD)
NPI score	0.87 (2.94)	3.08* (4.69)	12.28**^,#^ (13.29)
Zarit score	2.32 (3.96)	3.92 (6.05)	20.11**^,#^ (17.38)

SD: Standard Deviation;

* Mann-Whitney test-controls versus CIND: U=693.500, p=0.002;

** Mann-Whitney test – controls versus AD: NPI (U=690.000; p<0.001) and Zarit (U=405.500; p<0.001);

# Mann-Whitney – CIND versus AD: NPI (U=378.500; p<0.001) and Zarit (U=198.000; p<0.001)

Of the 61 AD patients included in the present study, 25 (41%) were classified as mildly demented (CDR=1), 29 (47.5%) moderately demented, and 6 (9.8%) as severely demented. To compare the groups, Mild AD was considered CDR 1, and Moderate and Severe AD as CDR 2 and CDR 3,respectively, but no statistically significant differences were observed among the groups.

When evaluating groups using the Spearman rank correlation coefficient, a significant positive correlation was observed between the NPI and Zarit scores (Spearman=0.684; p<0.001). Stratifying subjects by diagnosis, the positive correlation between NPI and Zarit remained strong for both CIND (Spearman=0.606; p=0.002) and AD (Spearman=0.589; p<0.001) groups, but was weak for controls (Spearman=0.300; p=0.008).

## DISCUSSION

In the present study, subjects with AD had mean scores on the Zarit and NPI of 20.11 and 12.28 points, respectively, scoring higher than controls. Moscoso et al.,^22^ in thirty-one elderly patients with AD from the outpatient unit of CEREDIC (Clinics Hospital – Cognitive Reference Center), observed a mean Zarit score of 31.77 and NPI of 34.97, scores higher than those observed in our study. Godinho et al.,^23^ studying a sample of 64 clinical outpatients with Alzheimer's disease, observed a mean NPI score of 35. One possible explanation for this difference is that our sample was community-based, while Moscoso et al.^22^ evaluated a clinical sample. Considering the CIND subjects' neuropsychiatric symptoms, population-based studies have shown that these symptoms are more frequent among this patient group compared with healthy elderly.^24,25^

Regarding the severity of dementia, there were no significant differences in caregiver burden scores by dementia group. According to Moscoso et al.,^22^ there is no consensus on the influence of the severity of dementia on caregiver stress.

Considering the mean Zarit scores for each NPI domain in Table 2, we found that Delusions, Hallucinations, Euphoria, Disinhibitions and Aberrant Motor Behavior were the domains with higher Zarit scores. Additionally, we observed a good correlation between the scores on the NPI and Zarit inventories. Moscoso et al.^22^ also observed a significant association between caregiver burden and total NPI scores. Several studies have observed a relationship between neuropsychiatric symptoms and caregiver stress. Fialho et al.^26^ evaluated 83 caregivers of patients diagnosed with dementia in the state of Minas Gerais, Brazil, and found a positive correlation between NPI and Zarit scores. In a review of studies from different regions of the world (North America, Europe/Australia and Asia) a significant association between neuropsychiatric symptoms and caregiver burden was reported.^26^ Moreover, the incidence of behavioral problems seems to have a higher potential to cause stress than persistence of these symptoms,^28^ but constant caregiving may significantly increase the risk of caregiver stress and burden.^29^

In conclusion, several factors influence the presence of caregiver burden, with neuropsychiatric symptoms showing a significant association with higher rates of caregiver stress, mainly related to symptoms such as aggression and delusions. Our study found a significant association between Zarit and NPI scores while subjects who presented one or more symptoms, such as delusions, hallucinations, euphoria, disinhibition and aberrant motor behavior, had the highest scores on the Zarit inventory. The adequate treatment and management of these neuropsychiatric symptoms in patients with dementia can have a significant impact on the quality of life of patients and their caregivers.
